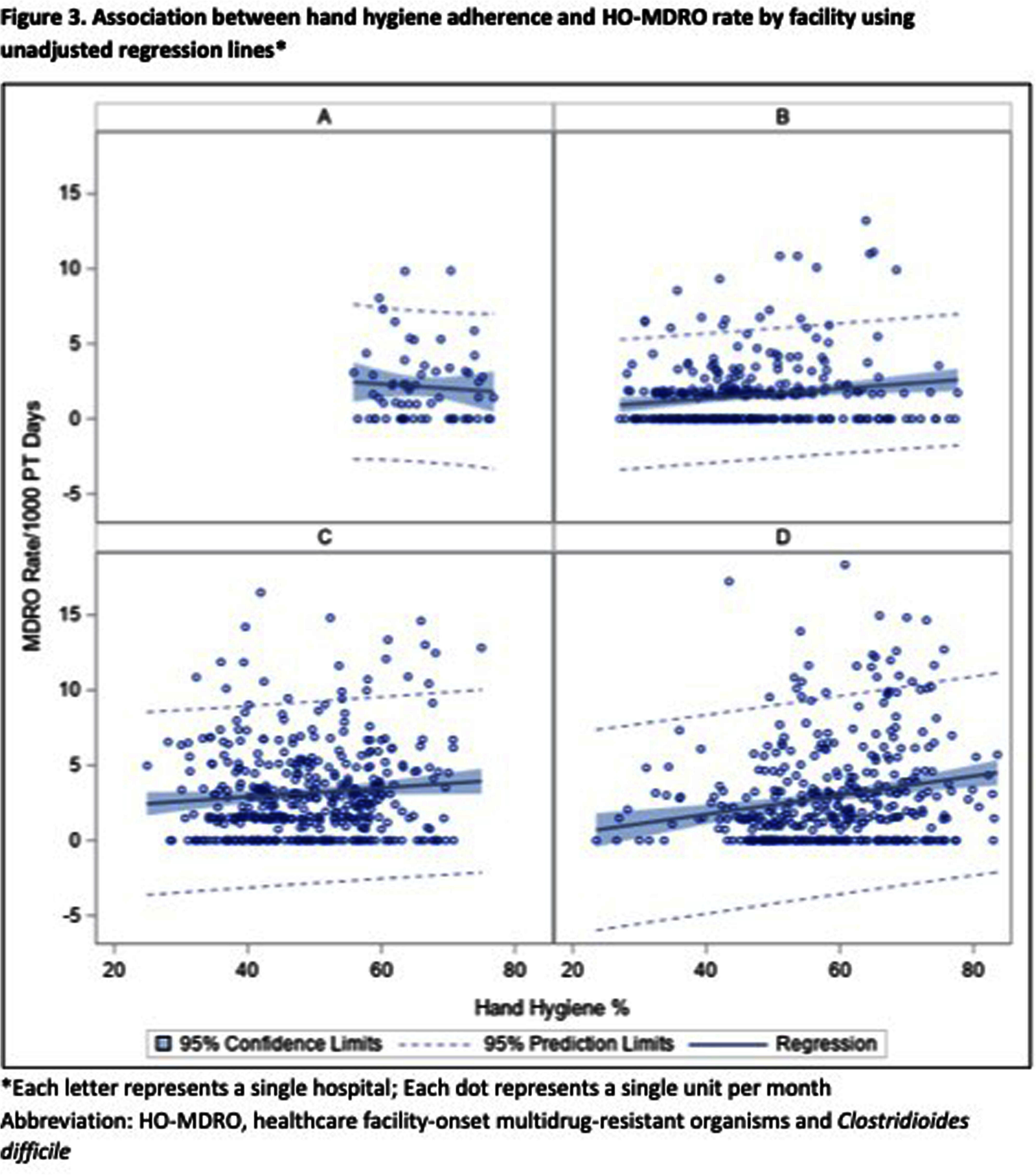# Relationship between Hand Hygiene and MDRO Acquisition after Implementation of an Electronic Hand Hygiene Monitoring System

**DOI:** 10.1017/ash.2024.248

**Published:** 2024-09-16

**Authors:** Radhika Prakash Asrani, Jesse Jacob, Chris Bower, James Steinberg, Patty Rider, Kari Love, Lindsey Gottlieb

**Affiliations:** Emory University, School of Medicine; Emory University; Emory University Hospital Midtown; Emory Saint Joseph’s Hospital; Emory Healthcare

## Abstract

**Background:** Hand hygiene (HH) is fundamental to preventing the transmission of pathogens between patients. Unfortunately, adherence to HH is suboptimal and monitoring adherence is challenging. Electronic HH monitoring systems (EHHMS) are emerging potential solutions to increase the number of HH observations and eliminate the potential for observation bias. This aim of this study is to assess the impact of improved HH adherence after the introduction of an EHHMS on the rates of healthcare facility-onset multidrug-resistant organisms and Clostridioides difficile (HO-MDRO). **Methods:** We performed a retrospective, quasi-experimental study to evaluate the impact of HH on HO-MDROs across 4 acute care facilities (59 hospital units, 14 of which were ICUs) from January 2021 - September 2022 after implementing an EHHMS in a large academic healthcare system. Clinical cultures from all sources were included; routine surveillance cultures were not collected during this period. HO determination was made using National Healthcare Safety Network (NHSN) definitions. The association between monthly unit-level HH adherence (%) and HO-MDRO rate per 1000 patient-days was assessed using mixed-effects Poisson regression using rate ratios (RR), which accounts for unobserved heterogeneity between units while controlling for number of tests ordered per month per hospital unit. HH adherence was stratified in quartiles (Q1: 24-43%, Q2: 43-51%, Q3: 52-61%, Q4: 61-84%). **Results:** During the study period, there were 23 million HH opportunities and 1875 MDROs in 772,930 patient-days. HH adherence increased from 41% January 2021 to 57% September 2022. ESBL, MSSA, and CDIFF accounted for most MDROs (Figure 1). The mean monthly HH adherence rate was 52% per unit, with a median of 1.66 (IQR: 0-3.5) MDROs/1000 patient-days. Mixed-effects Poisson regression suggested no significant overall relationship between HH adherence and MDRO rate (Figure 2). A close to null association was observed when comparing quartile two to quartile one (RR: 0.97, 95% CI: 0.82, 1.15), quartile three to quartile one (RR: 0.96, 95% CI: 0.79, 1.17), and quartile four to quartile one (RR: 1.05, 95% CI: 0.86, 1.28). Results were similar across hospitals (Figure 3). **Conclusions:** Although implementing an EHHMS led to an improvement in HH adherence, we were not able to demonstrate a resultant decrease in HO-MDROs. Potential explanations include the relatively rare outcomes of interest, unrecognized confounders, and the complex interaction between HH and HO-MDROs, since poor HH adherence on a unit may lead to increased attention from infection prevention and therefore increased focus on other MDRO prevention measures.

**Figure:**
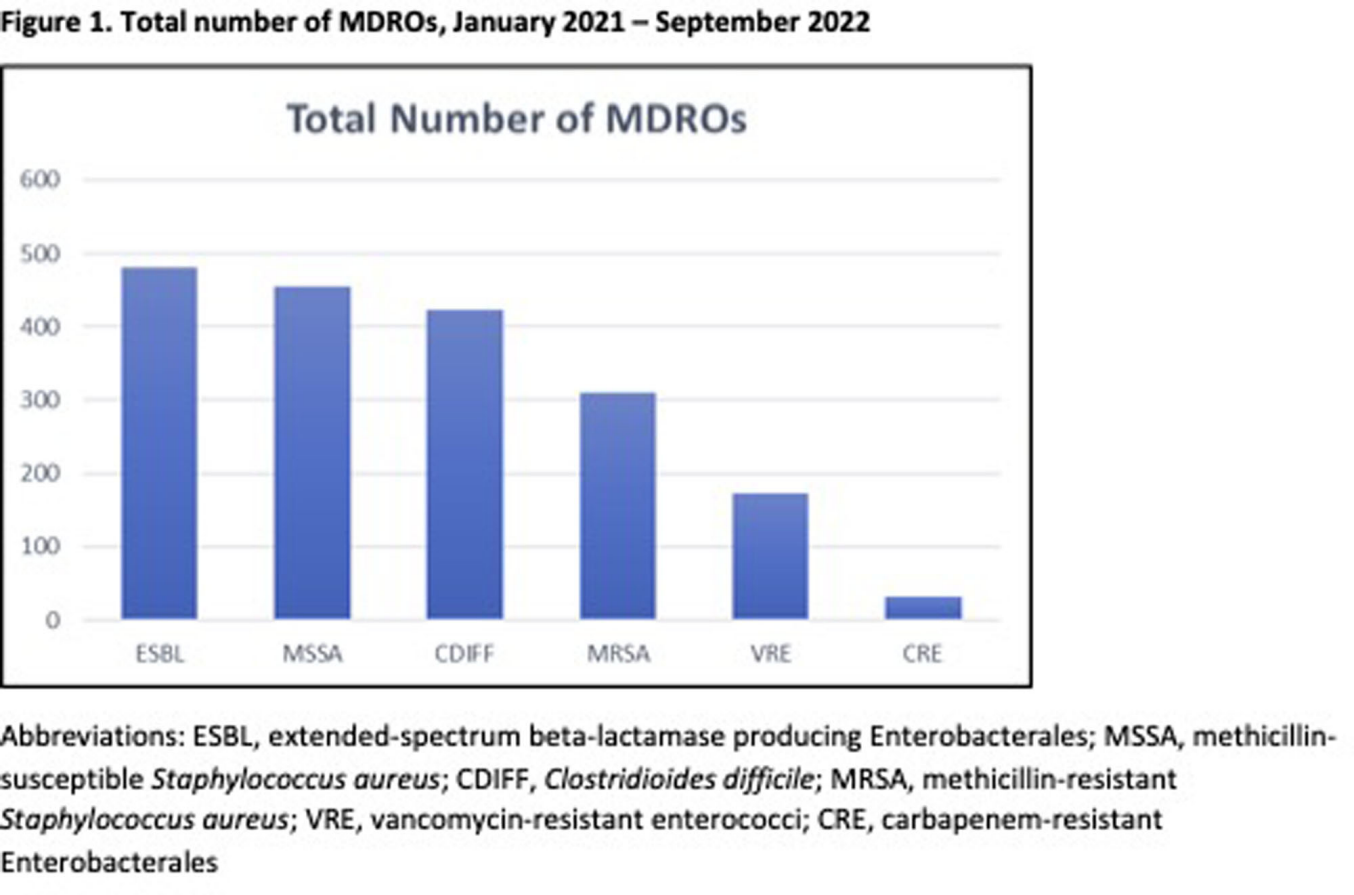

**Figure:**
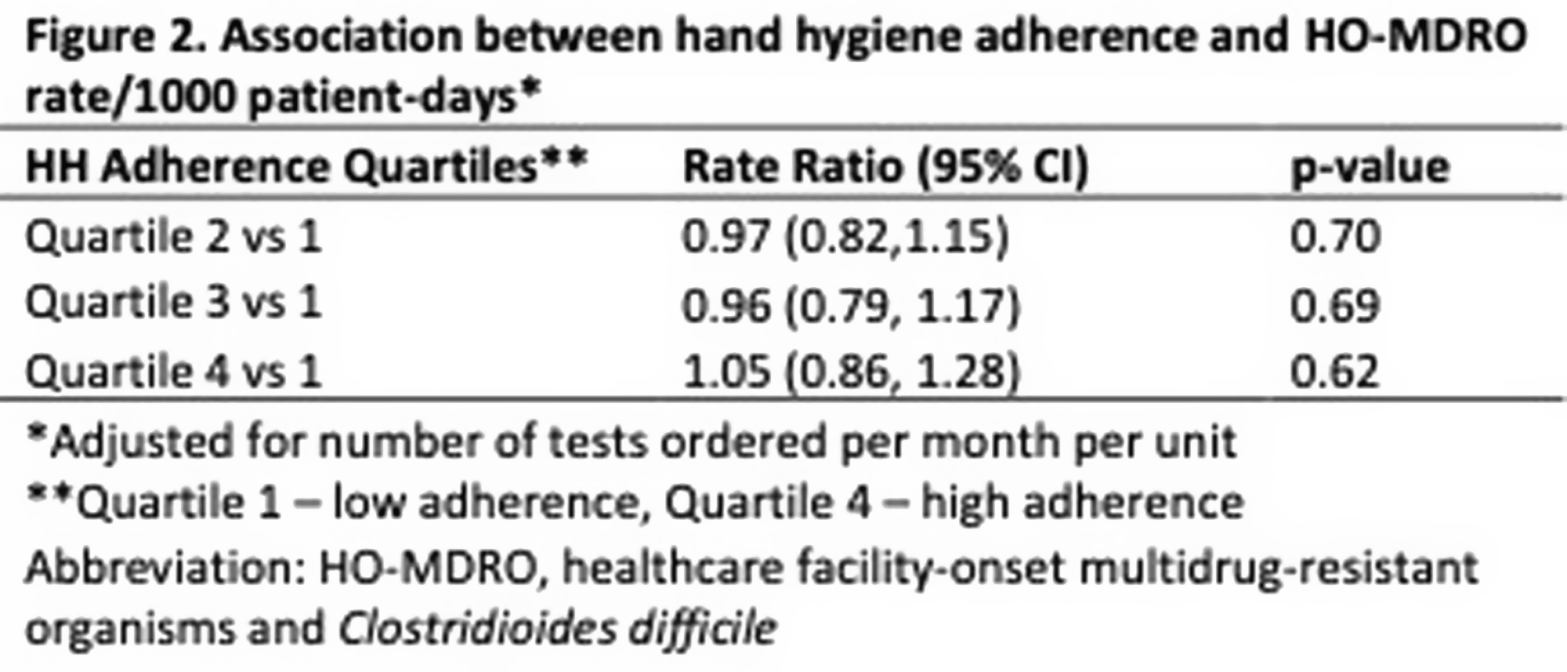

**Figure:**